# Peroxynitrite decomposition catalyst reduces vasopressin requirement in ovine MRSA sepsis

**DOI:** 10.1186/s40635-019-0227-4

**Published:** 2019-09-09

**Authors:** Osamu Fujiwara, Satoshi Fukuda, Ernesto Lopez, Yaping Zeng, Yosuke Niimi, Douglas S. DeWitt, David N. Herndon, Donald S. Prough, Perenlei Enkhbaatar

**Affiliations:** 1 Department of Anesthesiology, University of Texas Medical Branch, 301 University Boulevard, Galveston, TX 77555 1102 USA; 2 Shriners Hospital for Children, Galveston, Texas USA; 3 Department of Surgery, University of Texas Medical Branch, Galveston, Texas USA

**Keywords:** Peroxynitrite decomposition catalyst, WW-85, Arginine vasopressin, Septic shock, Vascular hypo-responsiveness, Refractory shock

## Abstract

**Background:**

Sepsis is one of the most frequent causes of death in the intensive care unit. Host vascular hypo-responsiveness to vasopressors during septic shock is one of the challenging problems. This study tested the hypothesis that adjunct therapy with peroxynitrite decomposition catalyst (WW-85) would reduce arginine vasopressin (AVP) requirements during sepsis resuscitation, using ovine sepsis model.

**Methods:**

Thirteen adult female Merino sheep, previously instrumented with multiple vascular catheters, were subjected to “two-hit” (cotton smoke inhalation and intrapulmonary instillation of live methicillin-resistant *Staphylococcus aureus*; 3.5 × 10^11^ colony-forming units) injury. Post injury, animals were awakened and randomly allocated to the following groups: (1) AVP: injured, fluid resuscitated, and titrated with AVP, *n* = 6 or (2) WW-85 + AVP: injured, fluid resuscitated, treated with WW-85, and titrated with AVP, *n* = 7. One-hour post injury, a bolus intravenous injection of WW-85 (0.1 mg/kg) was followed by a 23-h continuous infusion (0.02 mg/kg/h). Titration of AVP started at a dose of 0.01 unit/min, when mean arterial pressure (MAP) decreased by 10 mmHg from baseline, despite aggressive fluid resuscitation, and the rate was further adjusted to maintain MAP. After the injury, all animals were placed on a mechanical ventilator and monitored in the conscious state for 24 h.

**Results:**

The injury induced severe hypotension refractory to aggressive fluid resuscitation. High doses of AVP were required to partially attenuate the sepsis-induced hypotension. However, the cumulative AVP requirement was significantly reduced by adjunct treatment with WW-85 at 17–24 h after the injury (*p* < 0.05). Total AVP dose and the highest AVP rate were significantly lower in the WW-85 + AVP group compared to the AVP group (*p* = 0.02 and 0.04, respectively). Treatment with WW-85 had no adverse effects. In addition, the in vitro effects of AVP on isolated artery diameter changes were abolished with peroxynitrite co-incubation.

**Conclusions:**

The modulation of reactive nitrogen species, such as peroxynitrite, may be considered as a novel adjunct treatment option for septic shock associated with vascular hypo-responsiveness to vasopressors.

## Background

In the USA, the annual incidence of sepsis is around 750,000, causing approximately 225,000 deaths, and these numbers were recently reported to be rising [1–4]. The development of septic shock has been reported to increase the mortality of these patients up to 70% [4–7]. Historically, sepsis has mainly been caused by gram-negative bacteria. However, antibiotic-resistant gram-positive bacteria, such as methicillin-resistant *Staphylococcus aureus* (MRSA), are becoming a frequent source of sepsis [4, 8].

There is no specific therapy for sepsis and septic shock. Supportive therapy includes aggressive fluid resuscitation, early administration of antibiotics, and use of vasopressors such as norepinephrine (NE) [9]. Arginine vasopressin (AVP) has been advocated to be used as an adjunct therapy for hypotension refractory to NE during septic shock [9, 10]. However, its high doses increased the mortality of severe septic patients [11–14].

The vascular hypo-responsiveness to vasopressors, in septic shock, is a multi-factorial problem [15–18]. The pathophysiology of its occurrence is not fully investigated. One of the major mechanisms of the shock refractory to vasopressors is excessive production of nitric oxide (NO) and formation of its byproducts, such as peroxynitrite [19–21]. Peroxynitrite has been increasingly implicated to play a critical role in several aspects of sepsis and septic shock pathophysiology, including severe cardiovascular collapse [20–22]. Because NO synthase inhibitors may produce unwanted increases in pulmonary vascular resistance [23, 24], the modulation of reactive nitrogen species (RNS), such as peroxynitrite, may become an attractive approach to improve the host response to vasopressors.

In this study, we hypothesized that excessive formation of peroxynitrite may cause a vascular hypo-responsiveness to AVP, and its pharmacological modulation could potentially be beneficial in the treatment of MRSA-induced sepsis. To test our hypothesis, we have tested the effects of WW-85, a novel peroxynitrite decomposition catalyst, as an adjunct therapy to titrated AVP in our well-characterized ovine MRSA-induced sepsis model.

## Material and methods

### Animal care and use

Thirteen adult female Merino sheep (approximately 3 years of age and body weight [BW] 33.2 ± 1.9 kg) were used. In addition, small mesenteric arteries were isolated from three healthy sheep used for other studies as sham control.

The study was approved by the Institutional Animal Care and Use Committee (IACUC) of The University of Texas Medical Branch and conducted in compliance with the guidelines of the National Institutes of Health and the American Physiological Society for the care and use of laboratory animals. Animals were group-housed during a 14-day quarantine period at the Animal Research Center and placed in individual metabolic cages upon transferring to the Translational ICU (TICU) for study. Sheep were housed within sight of other sheep in a temperature/humidity controlled environment with dark/light cycles. Animals had free access to food (hay and pellets) and water until the start of the study. Sheep were chosen because of their close resemblance of the pathophysiologic and immune responses seen in humans with sepsis [25, 26].

### Surgical preparation

Fasted animals were anesthetized with ketamine (500 mg IM and 300 mg IV) (KetaVed™, VEDCO Inc., St. Louis, MO) and inhaled isoflurane (1–5 vol%) (Piramal Critical Care Inc. Bethlehem, PA), and multiple vascular catheters were surgically inserted (pulmonary arterial, femoral arterial, and left atrial), as described previously [25, 27]. After the surgical procedures, animals were awakened and monitored in the TICU in a conscious state for 5–7 days with free access to food and water. Pre- and post-surgical analgesia was provided with long-acting (72 h) Buprenorphine SR (0.05 mg/kg, SR Veterinary Technologies, Windsor, CO).

### Experimental protocol

After 5–7 days of recovery from surgical instrumentation, baseline (BL) variables were taken to ensure that the health condition in all studied animals was comparable (Table 1). Following the collection of BL values, a tracheostomy tube and urine catheter insertion was performed under ketamine and isoflurane anesthesia, and then sepsis was induced by a “two-hit” injury as described previously [25, 27]. In brief, anesthetized sheep received 48 breaths (four sets of 12 breaths per set) of cooled cotton smoke (< 39 °C) from a modified bee smoker via a tracheostomy tube. Arterial carboxyhemoglobin (aCO-Hb) levels were determined (CO-Oximeter IL 682; GMI, Ramsey, MN) after each set of smoke breaths. After completing the smoke insufflations, 3.5 × 10^11^ colony-forming units of live MRSA (strain; TCH1516 [USA300-HOU-MR], ATCC, Manassas, VA) was instilled in the lung by using a bronchoscope (model BF-P40; Olympus, Japan). Bacteria were suspended in 30 mL of saline and instilled into the right lower and middle lobes and the left lower lobe (10 mL each).

**Table 1:** Cardiovascular, respiratory, and biochemical variables during the baseline period and at 24 h of sepsis in two groups

Variables	Group	BL						
		0 h	3 h	6 h	9 h	12 h	18 h	24 h
Core body temperature (°C)	AVP	38.9 ± 0.1	40.1 ± 0.2	40.6 ± 0.1	40.3 ± 0.2	40.2 ± 0.1	40.1 ± 0.1	39.8 ± 0.2
	WW-85 + AVP	39.0 ± 0.1	40.0 ± 0.3	40.3 ± 0.3	40.1 ± 0.2	40.1 ± 0.2	40.3 ± 0.2	40.2 ± 0.2
Heart rate (beats/min)	AVP	85 ± 5	118 ± 14	151 ± 11	153 ± 12	135 ± 14	133 ± 10	130 ± 11
	WW-85 + AVP	93 ± 9	117 ± 8	134 ± 8	131 ± 11	123 ± 8	97 ± 7	100 ± 9
Mean arterial pressure (mmHg)	AVP	93.2 ± 1.7	96.3 ± 4.2	93.7 ± 2.9	88.2 ± 2.3	87.3 ± 3.0	94.0 ± 3.1	92.7 ± 4.7
	WW-85 + AVP	90.0 ± 1.2	90.3 ± 2.2	91.1 ± 2.0	89.7 ± 1.9	86.7 ± 1.9	91.7 ± 3.2	92.9 ± 3.0
Mean pulmonary artery pressure (mmHg)	AVP	18.2 ± 0.9	24.0 ± 1.2	25.3 ± 1.1	27.8 ± 1.5	29.7 ± 1.4	32.7 ± 1.9	31.8 ± 2.7
	WW-85 + AVP	19.0 ± 1.0	21.7 ± 1.3	23.7 ± 2.5	24.6 ± 1.2	27.0 ± 1.1	29.0 ± 1.9	27.6 ± 2.2
Pulmonary artery wedge pressure (mmHg)	AVP	9.5 ± 0.8	14.8 ± 0.9	15.2 ± 1.1	14.0 ± 0.9	14.5 ± 0.9	17.5 ± 0.7	18.0 ± 2.0
	WW-85 + AVP	10.4 ± 1.3	13.1 ± 1.2	12.6 ± 1.1	13.9 ± 1.6	15.7 ± 0.8	16.6 ± 1.8	14.3 ± 1.2
Central venous pressure (mmHg)	AVP	6.0 ± 0.7	8.7 ± 0.6	8.8 ± 1.0	10.3 ± 1.2	12.0 ± 1.4	14.7 ± 2.0	15.5 ± 1.7
	WW-85 + AVP	7.6 ± 0.8	7.4 ± 0.9	9.6 ± 1.8	10.3 ± 1.7	10.6 ± 1.9	11.9 ± 1.8	11.1 ± 1.6
Systemic vascular resistance index (dynes/sec/cm^5^/m^2^)	AVP	1270 ± 103	1187 ± 122	1172 ± 108	784 ± 100	731 ± 77	752 ± 76	764 ± 115
	WW-85 + AVP	1127 ± 106	1077 ± 118	1133 ± 153	1087 ± 141	1048 ± 148	1218 ± 233	1289 ± 267
PaO_2_/FiO_2_ ratio (mmHg)	AVP	487 ± 10	331 ± 58	336 ± 71	323 ± 71	260 ± 55	210 ± 34	190 ± 45
	WW-85 + AVP	496 ± 9	373 ± 33	402 ± 43	398 ± 49	360 ± 47	298 ± 52	264 ± 53
Oxygenation index	AVP	2.1 ± 0.1	3.7 ± 0.9	3.5 ± 0.7	4.9 ± 1.4	6.4 ± 1.8	12.3 ± 5.7	10.8 ± 3.1
	WW-85 + AVP	2.0 ± 0.1	2.8 ± 0.3	2.6 ± 0.3	2.9 ± 0.5	3.3 ± 0.6	7.0 ± 2.9	9.3 ± 4.3
Peak airway pressure (cmH_2_O)	AVP	20.3 ± 1.5	19.1 ± 1.0	19.7 ± 1.0	23.7 ± 2.3	25.8 ± 2.9	33.2 ± 5.2	31.7 ± 3.5
	WW-85 + AVP	19.6 ± 1.1	19.7 ± 1.0	19.4 ± 1.1	21.0 ± 1.3	20.9 ± 1.4	28.3 ± 3.1	28.3 ± 3.9
Plateau airway pressure (cmH_2_O)	AVP	18.0 ± 1.2	16.8 ± 0.7	16.7 ± 0.8	18.2 ± 1.8	21.5 ± 2.0	27.2 ± 4.8	27.8 ± 2.9
	WW-85 + AVP	17.0 ± 1.1	16.4 ± 1.0	16.1 ± 0.8	17.7 ± 1.2	17.6 ± 1.3	24.4 ± 2.8	25.4 ± 3.9
Lung static compliance (mL/cmH_2_O)	AVP	33.3 ± 3.0	35.9 ± 2.6	36.9 ± 3.5	35.3 ± 5.6	27.7 ± 4.3	22.8 ± 4.2	20.4 ± 3.6
	WW-85 + AVP	34.0 ± 3.4	35.5 ± 3.3	36.1 ± 3.1	32.6 ± 3.9	33.0 ± 3.7	23.3 ± 4.2	24.0 ± 4.7
SvO_2_ (%)	AVP	54.5 ± 3.9	67.9 ± 2.1	66.5 ± 3.4	66.7 ± 1.4	64.2 ± 4.9	65.2 ± 4.8	64.1 ± 4.9
	WW-85 + AVP	61.5 ± 2.1	74.8 ± 2.1	65.4 ± 2.7	65.5 ± 2.2	63.1 ± 2.1	54.4 ± 4.5	50.5 ± 5.3
Glucose (mg/dL)	AVP	55.0 ± 2.9	63.2 ± 2.5	40.2 ± 3.1	41.2 ± 4.9	37.2 ± 3.4	39.2 ± 4.5	35.0 ± 2.7
	WW-85 + AVP	67.8 ± 3.4	81.7 ± 11.9	46.7 ± 3.1	40.5 ± 2.3	43.0 ± 2.0	41.8 ± 4.8	41.8 ± 5.1
Hematocrit (%)	AVP	25.7 ± 1.1	26.7 ± 2.2	29.5 ± 2.0	27.3 ± 1.9	28.2 ± 2.3	27.2 ± 1.8	25.2 ± 1.7
	WW-85 + AVP	28.7 ± 1.3	25.7 ± 1.0	27.7 ± 1.4	26.4 ± 1.4	27.4 ± 1.7	27.3 ± 2.2	28.9 ± 1.7

The animal numbers in AVP groups is *n* = 6 and WW-85 + AVP group is *n* = 7. The data were analyzed by Two-way ANOVA repeated measurement with Bonferroni post-hoc test. Data are expressed as mean ± SEM. (* *p*<0.05 between two groups)

After the injury, animals were awakened, brought to the TICU, and mechanically ventilated (Servo 300; Siemens, Sweden AVEA; CareFusion, Yorba Linda, CA), and cardiopulmonary variables were monitored for 24-h in a conscious state. Pre and post-injury analgesia was provided with subcutaneous administration of long-acting Buprenorphine SR (0.05 mg/kg, SR Veterinary Technologies, Windsor, CO).

Two sheep were studied at a time, side-by-side, and randomly allocated to the following groups: (1) AVP: injured, fluid resuscitated, titrated with AVP, and treated with intravenous saline as a vehicle of WW-85, *n* = 6 or (2) WW-85 + AVP: injured, fluid resuscitated, titrated with AVP, and treated with WW-85, *n* = 7. The WW-85 is an iron-pyridyl-porphyrin, substituted with a benzoic acid group (Radikal Therapeutics Inc.; West Tisbury, MA). This agent accelerates peroxynitrite degradation by acting as a catalyst for the isomerization of peroxynitrite to primarily nitrate [20, 28]. The initial WW-85 bolus was delivered intravenously (via jugular vein) at 1-h post injury with a dose of 0.1 mg/kg in 10 mL of saline, which was followed by continuous infusion (0.02 mg/kg in 250 mL of saline) for the remainder of the 24-h experimental period.

### Mechanical ventilation

All animals were mechanically ventilated using a volume-controlled ventilation mode, with a tidal volume (TV) of 12 mL/kg, and a positive end-expiratory pressure of 5 cmH_2_O. Notably, sheep requires higher TV compared to humans due to a larger anatomical dead space and ratio of dead space to TV compared to humans [29]. Respiratory rate and fraction of inspired oxygen (FiO_2_) were initially set at 20 breaths/min and at 1.0 for the initial 3 h and further adjusted to keep PaCO_2_ between 30 and 40 mmHg and PaO_2_ at ~ 100 mmHg, respectively.

### Measured variables

Catheters were connected to a monitor (IntelliVue MP50; Philips Medical Systems, Andover, MA) via a pressure transducer (PX4X4; Edward Lifescience, Irvine, CA), and core body temperature (BT), mean arterial pressure (MAP), heart rate (HR), mean pulmonary arterial pressure (mPAP), left atrial pressure (LAP), and central venous pressure (CVP) were continuously monitored, as previously described [25, 27]. Cardiac output (CO) was determined intermittently by thermodilution techniques with cardiac output module (M1012A; Hewlett Packard, Santa Clara, CA). Cardiac index (CI) and systemic vascular resistance index (SVRI) were calculated using the formulas CI = CO/BSA and SVRI = 80 × (MAP − CVP)/CI [25, 27]. Sheep body surface area (BSA) was calculated using the equation BSA = 0.084 × (BW kg) 2/3 [30].

Arterial and venous blood gas samples were measured with a blood gas analyzer (GEM premier 3000; Instrumentation Laboratories, Lexington, MA) intermittently to determine pulmonary gas exchange, hematocrit, and blood lactate and glucose levels. The urine output was measured every 3 h, and cumulative fluid net balance was calculated as described previously [25, 27].

### Fluid resuscitation and vasopressor support

All animals were fluid resuscitated starting with an initial infusion rate of 2 mL/kg/h lactated Ringer’s solution (LR) (Baxter Healthcare Corporation, Deerfield, IL). Then, the LR rate was titrated every 3 h to maintain hematocrit close to BL levels ± 3%, as previously described [25, 27]. During the study period, the animals had free access to food but not to water to accurately determine fluid intake.

The MAP was recorded every hour, and AVP (Pitressin®, JHP Pharmaceuticals, LLC, Rochester, MI) was started with a dose of 0.01 unit/min when MAP dropped by 10 mmHg from the BL despite aggressive fluid resuscitation with LR. The MAP was continuously monitored and AVP rate was further adjusted stepwise (± 0.01 unit/min) every hour to keep MAP no more than 10 mmHg below BL.

### Euthanasia and tissue collection

After completion of the 24-h study period, the animals were euthanized with injection of ketamine (40 mg/kg), buprenorphine (0.01 mg/kg), and xylazine (3.0 mg/kg), following the IACUC-approved protocol and American Veterinary Medical Association Guidelines for Euthanasia [31]. Immediately after euthanasia, the organs and tissues were collected as previously described [25, 27].

### Isolation of small arteries and diameter measurement

Small arteries (diameter 200 μm) were isolated from the mesentery of three healthy sheep, and diameter was determined as previously described [32]. In brief, the small artery segments were mounted on a glass micropipette and bathed/perfused with warm (37.0 °C) oxygenated physiological salt solution. After 1 h of the artery diameter stabilization, using an inverted microscope equipped with a video camera and monitor, arterial intraluminal diameters were measured with intraluminal pressure of 20 mmHg. Vasoconstriction responses were determined and recorded following treatments: (1) adding AVP (69.9 nM), (2) adding peroxynitrite (product code 516620; EMD Millipore, Billerica, MS) (180 mM), and (3) adding a second dose of AVP (69.9 nM). The vessels were washed out for ~ 20 min to recover to their BL diameter after each treatment. The dosage of AVP and peroxynitrite were adopted from previously published studies [33–35].

### Statistical analysis

Statistical analysis was performed by two-way ANOVA repeated measurement with Bonferroni post hoc test, Mann-Whitney *U* test, or Wilcoxon matched-pairs signed-rank test using GraphPad Prism Version 8.0.1 (GraphPad Prism Software, Inc. San Diego, CA). The normality of the continuous data sets was analyzed by D’Agostino-Pearson’s omnibus K^2^ test. All data were reported as means ± SEM.

## Results

### Injury severity and survival

The severity of smoke injury was assessed by the highest aCO-Hb. There was no statistically significant difference in severity between the AVP group and WW-85 + AVP group (63.9 ± 13.8% vs. 61.3 ± 12.1%, respectively). All thirteen sheep in both groups were survived for the 24-h experimental period.

### MAP and AVP requirement

**Fig. 1:**
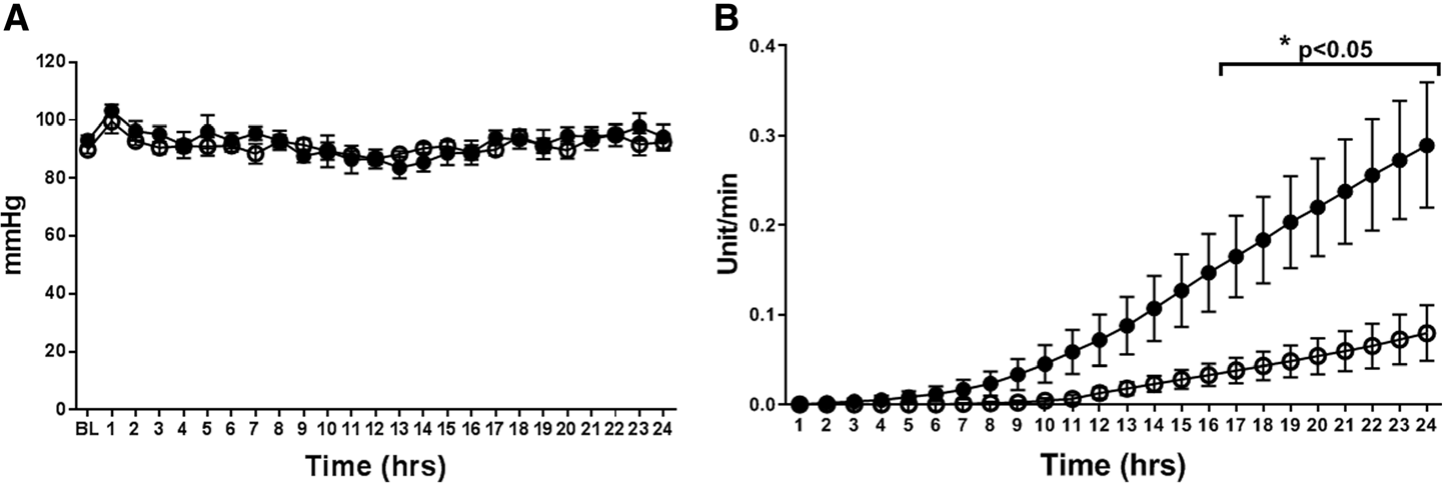
Mean arterial pressure (MAP) and cumulative requirement of arginine vasopressin (AVP) with or without peroxynitrite decomposition catalyst (WW-85) treatment on MRSA-induced septic shock. **a** Changes in MAP in the two groups. **b** Cumulative requirement of AVP to maintain MAP in the two groups. Closed circles represent the AVP group animals. Open circles represent the WW-85 + AVP group animals. Data are expressed as mean ± SEM (*p < 0.05 vs. AVP group)

**Fig. 2:**
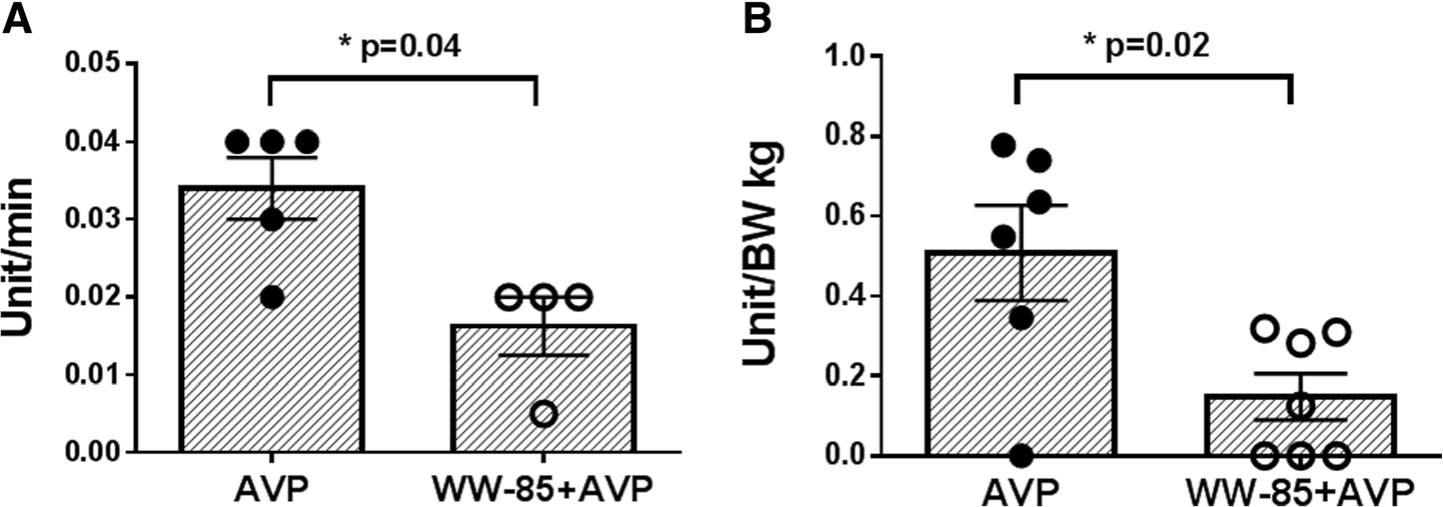
Effects of peroxynitrite decomposition catalyst (WW-85) on requirement of arginine vasopressin (AVP) in MRSA-induced septic shock. **a** The highest AVP rate (unit/min) in the two groups (*p* = 0.04 between the two groups). **b** Total AVP requirement (unit/BW kg) in the two groups (*p* = 0.02 between the two groups). Data are expressed as mean ± SEM

MAP was maintained with AVP support in both groups, and there were no significant differences in the two groups (Fig. 1a). However, the cumulative AVP requirements to keep MAP were significantly higher in the AVP group compared to the WW-85 + AVP group at 17–24 h after the injury (*p* < 0.05) (Fig. 1b). The start time of AVP administration was 7.6 ± 1.9 and 10.3 ± 1.2 h from the BL in the AVP and WW-85 + AVP groups, respectively. The highest AVP rate (unit/min) during the study period was significantly lower in the WW-85 + AVP group compared to the AVP group (0.016 ± 0.008 vs. 0.034 ± 0.009 unit/min, *p* = 0.04) (Fig. 2a). The total dose of AVP required for maintaining MAP was significantly higher in the AVP group compared to the WW-85 + AVP group (0.51 ± 0.29 vs. 0.15 ± 0.15 units/BW kg, *p* = 0.02) (Fig. 2b).

### Systemic hemodynamic changes

**Fig. 3:**
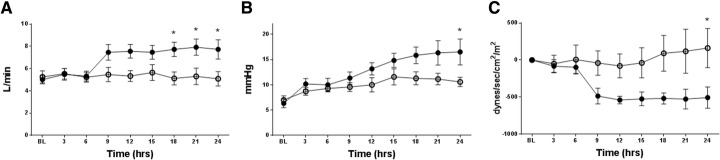
Cardiac output, left atrial pressure, and systemic vascular resistance index changes from the baseline in the two groups. **a** Cardiac output had significant differences between the two groups from 18 to 24 h post-injury (*p < 0.05 vs. AVP group). **b** Left atrial pressure had significant differences between the two groups at 24 h post-injury (*p < 0.05 vs. AVP group). **c** Systemic vascular resistance index changes from the baseline had significant differences between the two groups at 24 h post-injury (*p < 0.05 vs. AVP group). Closed circles represent the AVP group animals. Open circles represent the WW-85 + AVP group animals. Data are expressed as mean ± SEM

There were no significant differences found in BT, HR, MAP, mPAP, CVP, and SVRI between the two groups (Table 1). Cardiac output was significantly increased in the AVP group at 18–24 h after the injury compared to the WW-85 + AVP group (*p* < 0.05). Also, left atrial pressure was significantly higher in the AVP group compared to the WW-85 + AVP group at 24 h (*p* < 0.05) (Fig. 3a, b). SVRI decrements from the BL were significantly lower in the AVP group compared to the WW-85 + AVP group at 24 h after the injury (*p* < 0.05) (Fig. 3c).

### Fluid requirement and net fluid balance

**Fig. 4:**
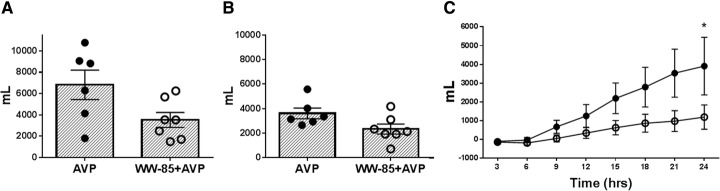
Total fluid input, total urine output, and net fluid balance in the two groups. **a** Total fluid input and **b** total urine output at 24 h had no significant differences between the two groups. **c** Net fluid balance had significant differences between the two groups at 24 h post-injury (*p* < 0.05). Closed circles represent the AVP group animals. Open circles represent the WW-85 + AVP group animals. Data are expressed as mean ± SEM

There were no significant differences in total fluid input and total urine output between the two groups (6813 ± 1384 vs. 3517 ± 702 mL and 3611 ± 437 vs. 2326 ± 409 mL, respectively) (Fig. 4a, b). However, the net fluid balance was significantly reduced in the WW-85 + AVP group at 24 h after the injury compared to the AVP group (*p* < 0.05) (Fig. 4c).

### Plasma protein concentration

**Fig. 5:**
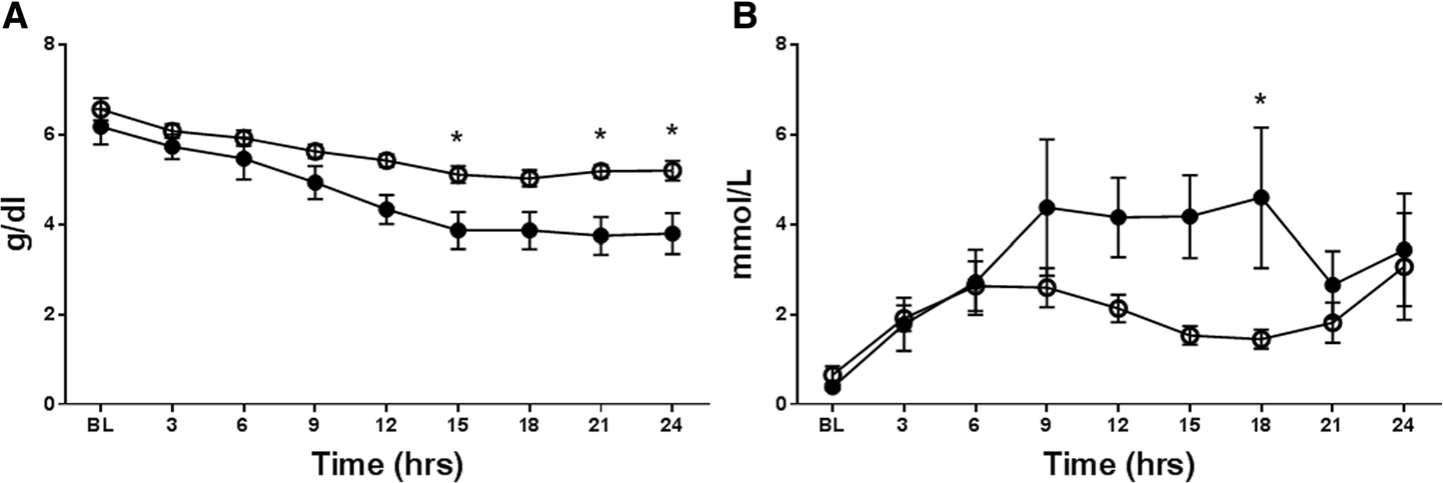
Plasma protein concentration and blood lactate concentration in two groups. **a** Plasma protein concentration had significant differences between two groups at 15 h and 21–24 h post-injury (*p* < 0.05). **b** Blood lactate concentration had significant differences between the two groups at 18 h post-injury (*p* < 0.05). Closed circles represent the AVP group animals. Open circles represent the WW-85 + AVP group animals. Data are expressed as mean ± SEM

Plasma protein concentration was significantly decreased in the AVP group compared to the WW-85 + AVP group at 15 and 21–24 h after the injury (*p* < 0.05) (Fig. 5a).

### Blood biochemical variables

Blood lactate concentration was significantly higher in the AVP group compared to the WW-85 + AVP group at 18 h after the injury (*p* < 0.05) (Fig. 5b). Blood glucose levels were comparable in both groups (Table 1).

### Gas exchange, pulmonary mechanics, and mixed venous oxygen saturation changes

Despite the tendency of the higher PaO_2_/FiO_2_ ratio in the WW-85 + AVP group compared to the AVP group, there were no significant differences in the lung oxygenation parameters (shunt fraction and oxygenation index) and pulmonary mechanics (peak, plateau, mean airway pressures, and lung static compliance) between the two groups (*p* > 0.05). SvO_2_ was comparable in both groups (Table 1).

### Isolated small artery diameter

**Fig. 6:**
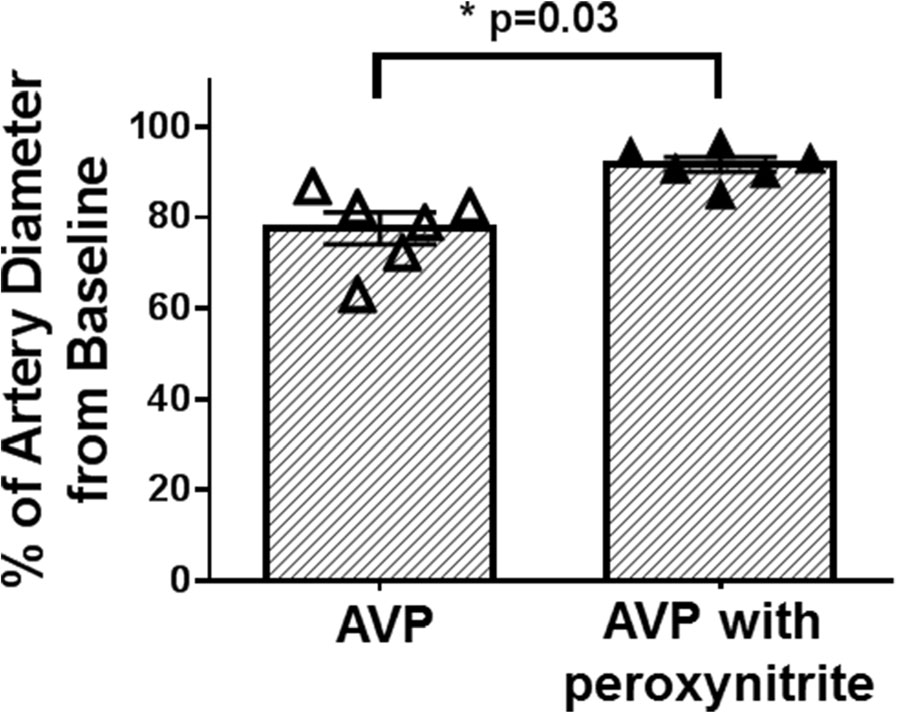
Isolated small artery diameter measurement. The contraction rate of small arteries (isolated from healthy sheep mesentery) after arginine vasopressin (AVP) administration was significantly reduced after peroxynitrite exposure compared to no peroxynitrite exposure (*p* = 0.03). Data are expressed as mean ± SEM

Initial treatment with AVP (without peroxynitrite exposure) reduced the diameter of isolated arteries to 77.5 ± 3.5% from the BL diameter. However, the response to AVP was blunted when the vessels were treated with peroxynitrite—the diameter of arteries was reduced only to 91.7 ± 1.6% (*p* = 0.03) (Fig. 6).

## Discussion

The main finding of this study was that the adjunct treatment with peroxynitrite decomposition catalyst WW-85 significantly reduced the requirements of AVP to maintain the MAP, suggesting that RNS such as peroxynitrite reduce the vascular response to AVP. An interesting finding was also that peroxynitrite modulation by WW-85 significantly reduced the net fluid balance and significantly attenuated plasma protein decreases and blood lactate increases.

Septic patients often develop severe hypotension refractory to both fluid resuscitation and vasopressors. Similarly, the septic sheep in this study required a large volume of fluid resuscitation. Administration of large volumes of fluid often augments the edema formation in soft tissues and vital organs and is associated with massive accumulation of fluid in the thoracic and abdominal cavities [36]. The resulting increases in abdominal or thoracic cavities pressure may be sufficient to restrict intracavitary organ perfusion. In addition, tissue edema increases the diffusion distance for oxygen, thus further impairing the microcirculation and augmenting tissue injury [37].

Intravascular volume depletion, as a result of external fluid loss and internal fluid redistribution, leads to severe hypotension, which is complicated by sepsis-induced vasodilatation. The latter is often refractory to the administration of vasopressors requiring higher volume resuscitation, which further aggravates edema formation and intracavitary fluid accumulation. Notably, prolonged use of high doses of vasopressors may be associated with higher mortality in patients with septic shock [11–14, 38, 39].

Although the mechanism(s) for refractory septic shock and vascular hyperpermeability has not been fully described, it can be related to multiple factors including the vasodilatory shock and impaired catecholamine receptors. Nitric oxide and its byproducts clearly play a major role in the pathophysiology of these conditions [15–17, 20–22, 38, 40]. Previously, we have demonstrated an important role of excessive NO in cardiopulmonary collapse and severe vascular leakage, by using various NO synthase (NOS) inhibitors, in ovine sepsis models [41–44]. We have also reported that expression of inducible NOS mRNA was peaked at 12 h after the injury sepsis onset [44]. Interestingly, in the present study, a sharp increase in AVP requirement was noted at the same time point (12 h) in the AVP group. Taken together, these results may suggest that excessive NO and its byproducts critically contributed to vascular hypo-responsiveness to AVP [45].

It is worth noting that NOS inhibitors, especially in high doses, potentially exert adverse effects when used clinically, resulting in controversy as to whether inhibition of NOS is beneficial or harmful in sepsis. Moreover, it is unclear which isoform of NOS represents the best target for clinical management. One alternative to inhibition of NO production is to more specifically reduce its downstream metabolites, such as peroxynitrite. At high concentrations, peroxynitrite is more toxic than NO and causes lipid peroxidation, protein oxidation and protein nitration, enzyme inactivation, and eventually cell necrosis [20]. Peroxynitrite has also been reported, in a few studies, to contribute to the loss of vascular contractility and fatal cardiovascular depression. Because peroxynitrite is formed regardless of which isoform of NOS is responsible for excessive NO production, the modulation of RNS such as peroxynitrite could be an attractive alternative for management of MRSA-induced sepsis [44, 46]. Thus, our aim was to investigate whether the adjunct therapy with peroxynitrite decomposition catalyst would improve the vascular response to AVP and effectively maintain MAP and reduce fluid retention. The results of our present study demonstrate that the adjunct treatment of peroxynitrite decomposition catalyst improved cardiovascular hemodynamics, such as cardiac output, left atrial pressure, and systemic vascular resistance index. Importantly, it attenuated the excess systemic fluid accumulation during sepsis.

Our present study has several limitations. First, we did not investigate the underlying mechanisms of how peroxynitrite caused the hypo-responsiveness to AVP. Thus, the precise mechanisms underlying salutary effects of adjunct treatment with peroxynitrite decomposition catalyst on cardiovascular responses to AVP remain not completely understood. However, based on our in vitro studies that peroxynitrite blunted the responses of isolated artery to AVP, we can speculate that RNS, such as peroxynitrite, may nitrosylate or nitrite V_1a_ receptors on the vascular smooth muscle, thus causing host hypo-responsiveness to AVP and eventually leading to septic shock. Further studies are warranted to explore these possible mechanisms. Secondly, we started the therapeutic intervention 1 h after the injury, which does not precisely mimic clinical scenario [47]. Also, we started the AVP titration at the early phase of hypotension (MAP > 65 mmHg) unlike a clinical scenario in which vasopressors are initiated when MAP is lower than 65 mmHg [1, 9, 47]. Thirdly, we did not use any supporting therapies for sepsis [47], such as norepinephrine (first-line vasopressor in sepsis) or antibiotics to investigate “pure” effects of testing compound without the interference of other factors. Another limitation is related to the relatively short study period (24 h), which did not consider the concomitant diseases or factors that are associated with human sepsis. Finally, we have used, for our in vitro studies, arteries isolated from only healthy sheep mesenterium.

Nevertheless, the model allowed us to most closely mimic the human hyperdynamic sepsis continuously monitoring cardiopulmonary hemodynamics in a conscious state without the effects of anesthetics. The study animals were given a standard of care, such as fluid resuscitation and positive ventilator support, in a similar way to what human patients would have in the ICU. Here, we report first time that modulation of peroxynitrite significantly reduced AVP requirements and improved net fluid balance using a well-characterized large animal translational model of sepsis and septic shock in an ICU setting.

## Conclusions

In conclusion, taken together, the results of our study demonstrate that modulation of peroxynitrite alleviates the severity of ovine MRSA-induced sepsis/septic shock by (1) attenuating severe hypotension by increasing the host vascular response to AVP and (2) reducing net fluid balance. Overall, the adjunct modulation of RNS, such as peroxynitrite to vasopressors, may be considered as a novel and efficient therapeutic option for treatment of septic shock patients. This approach is especially provocative as peroxynitrite is the product of excessive NO regardless of which NOS isoform is involved, and the major debate—whether the use of NOS inhibitors in the management of sepsis is beneficial—still remains. The approach is also of particular importance because the adjunct peroxynitrite modulation reduces the vasopressin requirement, thus preventing its unwanted adverse effects.

## Abbreviations

aCO-Hb: Arterial carboxyhemoglobin
AVP: Arginine vasopressin
BL: Baseline
BSA: Body surface area
BT: Core body temperature
CI: Cardiac index
CO: Cardiac output
CVP: Central venous pressure
FiO_2_: Fraction of inspired oxygen
HR: Heart rate
IACUC: Institutional Animal Care and Use Committee
LAP: Left atrial pressure
LR: Lactated Ringer’s solution
MAP: Mean arterial pressure
mPAP: Mean pulmonary arterial pressure
MRSA: Methicillin-resistant *Staphylococcus aureus*
NE: Norepinephrine
NO: Nitric oxide
NOS: Nitric oxide synthase
RNS: Reactive nitrogen species
SVRI: Systemic vascular resistance index
TICU: Translational Intensive Care Unit
TV: Tidal volume
